# Plant Growth Retardation and Conserved miRNAs Are Correlated to Hibiscus Chlorotic Ringspot Virus Infection 

**DOI:** 10.1371/journal.pone.0085476

**Published:** 2013-12-30

**Authors:** Ruimin Gao, Zi Yi Wan, Sek-Man Wong

**Affiliations:** 1 Department of Biological Sciences, National University of Singapore, Singapore; 2 Temasek Life Sciences Laboratory, Singapore; 3 National University of Singapore Suzhou Research Institute, Jiangsu, China; Key Laboratory of Horticultural Plant Biology (MOE), China

## Abstract

Virus infection may cause a multiplicity of symptoms in their host including discoloration, distortion and growth retardation. Hibiscus chlorotic ringspot virus (HCRSV) infection was studied using kenaf (*Hibiscus cannabinus* L.), a non-wood fiber-producing crop in this study. Infection by HCRSV reduced the fiber yield and concomitant economic value of kenaf. We investigated kenaf growth retardation and fluctuations of four selected miRNAs after HCRSV infection. Vegetative growth (including plant height, leaf size and root development) was severely retarded. From the transverse and radial sections of the mock and HCRSV-infected kenaf stem, the vascular bundles of HCRSV-infected plants were severely disrupted. In addition, four conserved plant developmental and defence related microRNAs (miRNAs) (miR165, miR167, miR168 and miR171) and their respective target genes *phabulosa* (*PHB*), auxin response factor 8 (ARF8), *argonaute 1* (*AGO1*) and *scarecrow-like* protein *1* (*SCL1*) displayed variation in expression levels after HCRSV infection. Compared with the mock inoculated kenaf plants, miR171 and miR168 and their targets *SCL1* and *AGO1* showed greater fluctuations after HCRSV infection. As HCRSV upregulates plant *SO* transcript in kenaf and upregulated *AGO1* in HCRSV-infected plants, the expression level of *AGO1* transcript was further investigated under sulfite oxidase (SO) overexpression or silencing condition. Interestingly, the four selected miRNAs were also up- or down-regulated upon overexpression or silencing of SO. Plant growth retardation and fluctuation of four conserved miRNAs are correlated to HCRSV infection.

## Introduction

 Hibiscus chlorotic ringspot virus (HCRSV) belongs to the genus *Carmovirus*, family *Tombusviridae* [1]. It is an icosahedral, positive sense single-stranded RNA plant virus. It has a (+)-sense ss RNA of 3911 nt, containing seven open reading frames (ORFs). HCRSV has been reported to cause systemic infection in *Hibiscus cannabinus* (kenaf), *H. sabdariffa* and *H. trionum*, and results in chlorotic local lesions on several members of *Chenopodium* spp. [1-5]. Successful infection with HCRSV results in severe symptoms and stunted growth on kenaf [2]. Symptoms have been characterized to be variable; however, chlorotic ringspots on the leaves of infected plants are considered the most characteristic symptom of HCRSV infection [1]. Other symptoms include mosaic patterns, rings, vein-clearing and vein-banding [6]. Regarding the host-virus interaction, HCRSV coat protein (HCRSV-CP) interacts with sulfite oxidase (SO) [7] which in turn, triggers sulfur enhanced defense (SED) and leads to enhanced plant resistance [8]. 

 MicroRNAs (miRNAs) are small RNAs (~22 nucleotides) which are derived from non-protein-coding RNAs and they negatively regulate gene expression [9,10]. Many miRNAs, with their syntheses and regulation affected by the signals generated from environmental stress, are conserved in plants [11]. MiRNAs are involved in plant development, signal transduction, protein degradation and response to environmental stress and pathogen invasion [12-15]. The targets of most plant miRNAs are transcription factors which play important roles in plant defense responses [16,17]. It is well studied that the targets of miR165, miR167, miR168 and miR171 are *phabulosa* (*PHB*), auxin response factor 8 (ARF8), *argonaute 1* (*AGO1*) and *scarecrow-like* protein *1* (*SCL1*) respectively, playing essential roles in regulating plant development [18-21]. Furthermore, miRNAs are known to modulate plant viral diseases [22,23]. After virus infection, plant conserved miR165, miR167, miR168 and miR171 showed different expression levels at different stages [20]. Thus, miR165, miR167, miR168 and miR171 were selected for investigation in this study. 

 Among the targets of four selected miRNAs, *AGO1* was known to be involved in the feedback regulation of miRNAs [24,25]. Furthermore, since AGO1 is the key component of RNA induced silencing complex (RISC), it plays essential roles in the small RNA induced silencing pathway [26]. Downregulation of some host genes have been speculated to elicit disease symptoms [27]. Indeed, disease symptoms caused by Cucumber mosaic virus (CMV) satellite RNA are the consequence of siRNA-directed RNA silencing of chlorophyll gene CHLI [28,29]. Furthermore, plant developmental abnormalities were caused through misregulation of the miR167 targeting ARF [21]. 

 As a non-wood fiber producing crop, the reduction in fiber yield of kenaf is considered to be of economic significance. In this study, we compared the gross morphology and cross sections of the stem in mock and HCRSV-infected kenaf plants. Since plant growth retardation was related to certain plant developmental genes expression profiles which are regulated by miRNAs, the four selected plant conserved developmental related miRNAs (miR165, miR167, miR168 and miR171) were investigated after HCRSV infection. This study showed that the plant developmental related miRNAs fluctuated after HCRSV infection, which displayed plant growth retardation. Analyzing the plant developmental related miRNAs will improve the understanding of viral infection and present strategies to prevent infection in future.

## Materials and Methods

### 1: Plant materials and growth condition

 Kenaf seeds (cultivar Everglades 41) were obtained from Mississippi State University, USA) and germinated on potting mixture (Tref, Netherlands) for 7 days. Kenaf seedlings were transferred into the same potting mixture after emergence of true leaves. All plants were grown in a plant growth room under 16 h light and 8 h dark at 25 °C with fluorescent tube (Model:CS-22, FoFo Shan Light, China).

### 2: Plasmid construction

 The *SO* coding region was subcloned from pSAT6-cEYPF-C1-SO vector into binary vector of 35SpGreen [30,31] to form a construct 35SpGreen-SO. For the SO silencing study, artificial-miRNA amiRSO was engineered into the miR319a precursor (plasmid pRS300) by site-directed mutagenesis, following the protocol by Rebecca Scheab Max-Plank Institute for Developmental Biology, Tuebingen, Germany (2005) (http://wmd.weigelworld.org/cgi-bin/mirnatools.pl. Ossowski Stephan, Fitz Joffrey, Schwab Rebecca, Riester Markus and Weigel Detlef, personal communication). The artificial miRNAs amiRSO was also cloned into the pGreen vector. Both of the overexpression and silencing vectors were transferred into *Agrobacterium tumefaciens* GV3101 using electroporation and the transformed colonies were verified by colony PCR and sequencing.

### 3: Staining of transverse and radial sections from mock and HCRSV-infected plants

 Young stems were flash frozen with liquid nitrogen. The frozen stems of mock and HCRSV-infected kenaf plants were cut into 50-60 µm thin sections using Leica Cryostat Model 1850. The slices were transferred to glass slides, stained in a drop of 0.05% aqueous toluidine blue O for 3 minutes and washed with 30% glycerol. After drying on the slides, the stained sections were observed using a light microscope (Model DP72, Olympus, USA). 

### 4: Virus inoculation

 Before virus inoculation, young seedlings at the cotyledon stage were verified to be healthy and virus-free. Fully developed young leaves from HCRSV-infected kenaf plants exhibiting chlorotic spots (0.1 g) were homogenized in 0.5 ml of virus inoculation buffer (0.01 M potassium phosphate buffer, pH 7.0) (using Protocol online at http://www.protocol-online.org/biology-forums/posts/16381.html) and used for inoculation. Mock-inoculation was carried out by rubbing inoculation buffer alone onto the cotyledons. Fully-developed young leaves were harvested at 3, 6, 9, 12, 15, 20, 25 and 30 days post inoculation (dpi) to quantify the transcript levels of miRNAs and their respective target genes. This experiment was carried out thrice.

### 5: RNA extraction and cDNA synthesis

 Fully-developed young leaves showing severe symptoms were collected for RNA extraction at previously mentioned time points post-inoculation with HCRSV. RNA was extracted using TRIzol^®^ reagent (Invitrogen, USA). The integrity and quality of the extracted RNA were determined by non-denaturing agarose gel electrophoresis. Total RNA (~2 μg) was used to generate cDNAs through reverse transcription, using oligo(dT)_15_ or specific stem-loop primers (Table 1) [20] and SuperScript^®^ III Reverse Transcriptase kit (Invitrogen, USA). 

**Table 1:** Primers used in this study.

Primers	Sequences (5’ to 3’)
miR165-RT	CTCAGCGGCTGTCGTGGACTGCGCGCTGCCGCTGAGGGGGRATG
miR165-F	CTGTGTCGGACCAGGCTTC
miR165-R	GGCTGTCGTGGACTGCG
miR167-RT	GCGTGGTCCACACCACCTGAGCCGCCACGACCACGCTAGATCAT
miR167-F	CGTGCGTGAAGCTGCCA
miR167-R	TCCACACCACCTGAGCCG
miR168-RT	CTCAGCGGCTGTCGTGGACTGGGTGCTGCCGCTGAGTTCCCGAC
miR168-F	CGTGTGTCGCTTGGTGCA
miR168-R	GGCTGTCGTGGACTGGGTG
miR171-RT	GCGTGGTCCACACCACCTGAGCCGCCACGACCACGCGATATTGG
miR171-F	CGTGCGTGATTGAGCCGT
miR171-R	TCCACACCACCTGAGCCG
I. amiR-So F	GATTTTAATTGACTGCACGTCTTTCTCTCTTTTGTATTCC
II. amiR-So R	GAAAGACGTGCAGTCAATTAAAATCAAAGAGAATCAATGA
III. amiR-SO* F	GAAAAACGTGCAGTCTATTAAATTCACAGGTCGTGATATG
IV. amiR-SO* R	GAATTTAATAGACTGCACGTTTTTCTACATATATATTCCT
A	CTGCAAGGCGATTAAGTTGGGTAAC
B	GCGGATAACAATTTCACACAGGAAACAG
Hc.qARF8F312	ATCGTTGTGTCCTGAAAGCAG
Hc.qARF8R464	ACTTGTGGAACTGATGTTGAGC
Hc.qPHBF14	CGGATTCTATTGGCATTGTTGC
Hc.qPHBR151	ATCGGCAGTCACGAAACCA
Hc.qAGO1F375	ATGTCACCCATCCTCACCCT
Hc.qAGO1R481	AGCACAAACCAGACCAGCA
Hc.qSCL1F403	TCGTAAAGCAGTTGTCACCCA
Hc.qSCL1R537	TCACAGCATCAAGGGACTCG
HcAct-qF603	ACGAGCAGGAACTGGAGACT
HcAct-qR734	TGAGTGATGGCTGGAAGAGGA
ORFHcAGO1F	CTCTTTCTCTGCGTAGTGACTGGTGACT
ORFHcAGO1R	TAGCGAGGAACGATAAAGGCTTGTA

### 6: Gene expression analysis of four conserved miRNAs and their respective target genes using qRT-PCR

 Expression levels of selected transcripts were analyzed via quantitative real time reverse-transcription PCR (qRT-PCR) after cDNA synthesis. The qRT-PCR was set up in a total volume of 5 μl, and was carried out in the CFX384^TM^ real-time PCR detection system (Bio-Rad, USA). Actin gene was used as the internal control, which showed similar expression profiles in the mock and HCRSV-infected plants. In addition, no difference was observed for actin gene expression in healthy and agro-infiltrated kenaf leaves, respectively. The expression levels of the miRNAs were detected following previously described protocols [20,32]. The experiment was carried out thrice. Each test consisted of three biological repeats and each sample contained three technical repeats. The relative gene expression amount was analyzed using the 2^-∆∆C^_T_ method. The values of miRNAs and their respect target genes in HCRSV-infected plants were calculated by subtracting the values from mock controls which were all set to 1 for standardization. Means of three independent biological repeats were shown with standard deviations. 

### 7: *Agrobacterium*-mediated transient expression

*Agrobacterium tumefaciens* liquid cultures containing each of the pGreen-SO, pGreen-amiRSO plasmids and 35SpGreen (negative control) plasmids, were grown to an OD reading (at 600 nm) between 1.0-1.5 and harvested. The cell pellet was resuspended in infiltration buffer (pH 7) containing 10 mM each of MgCl_2_ and 2-(N-morpholino) ethanesulfonic acid (MES), and 100 µM acetosyringone and infiltrated into kenaf leaves using a syringe without needle [6]. The selected kenaf leaves used for agro-infiltration were taken from the youngest fully expanded leaves at 14 days after seed emergence. The infiltrated regions were cut out and used for RNA extraction. The experiment was carried out thrice. The relative gene expression amount was analyzed using the 2^-∆∆C^_T_ method. Significant differences among silenced-SO, mock-infiltration and overexpressed-SO were calculated using the one sample Student’s *t*-test analysis.

### 8: Cloning of four conserved miRNAs and their respective target genes

 Since the conserved miR165, miR167, miR168 and miR171 were not verified in *H. cannabinus* previously, stem-loop reverse primer (Table 1) was used to carry out the reverse transcription. The PCR products of the four conserved miRNAs were cloned into pGEM TA vector (Promega, USA) for sequencing and verification. 

 The *H. cannabinus PHB*, *ARF8*, *AGO1* and *SCL1* genes were not available in the GenBank database and were thus amplified and sequenced in this study. Using degenerate primers, the four target genes were amplified from kenaf. Using the DNAMAN v5.0 sequence analysis program, multiple-sequence alignment (MSA) was performed with four different miRNA target genes. A set of forward and reverse degenerate PCR primers were designed based on conserved sequences. The PCR primers were tested using cDNAs obtained from kenaf plants. The PCR products containing the conserved sequences from each of the four genes were cloned into the pGEM TA Vector (Promega, USA) and sequenced. The qRT-PCR primers (Table 1) were designed accordingly using the gene sequences obtained. 

### 9: RACE PCR to amplify the complete sequence of *AGO1* from kenaf

 Based on the conserved sequence of *AGO1* gene, RACE kit GeneRacer^TM^ kit (Invitrogen, USA) was used to amplify the respective 5’- and 3’-sequences. Nested PCR was performed to amplify the specific PCR product. The 5’- and 3’-PCR products obtained were cloned into pGEM TA Vector (Promega, USA) and sequenced. After the sequences were blasted with PUBMED and confirmed, a pair of primers complementary to each of the 5’- and 3’-termini were designed and used to amplify the complete ORF of the *AGO1*. All primers used were listed in Table 1. 

## Results

### 1: Morphology of HCRSV-infected kenaf plants

**Figure 1:**
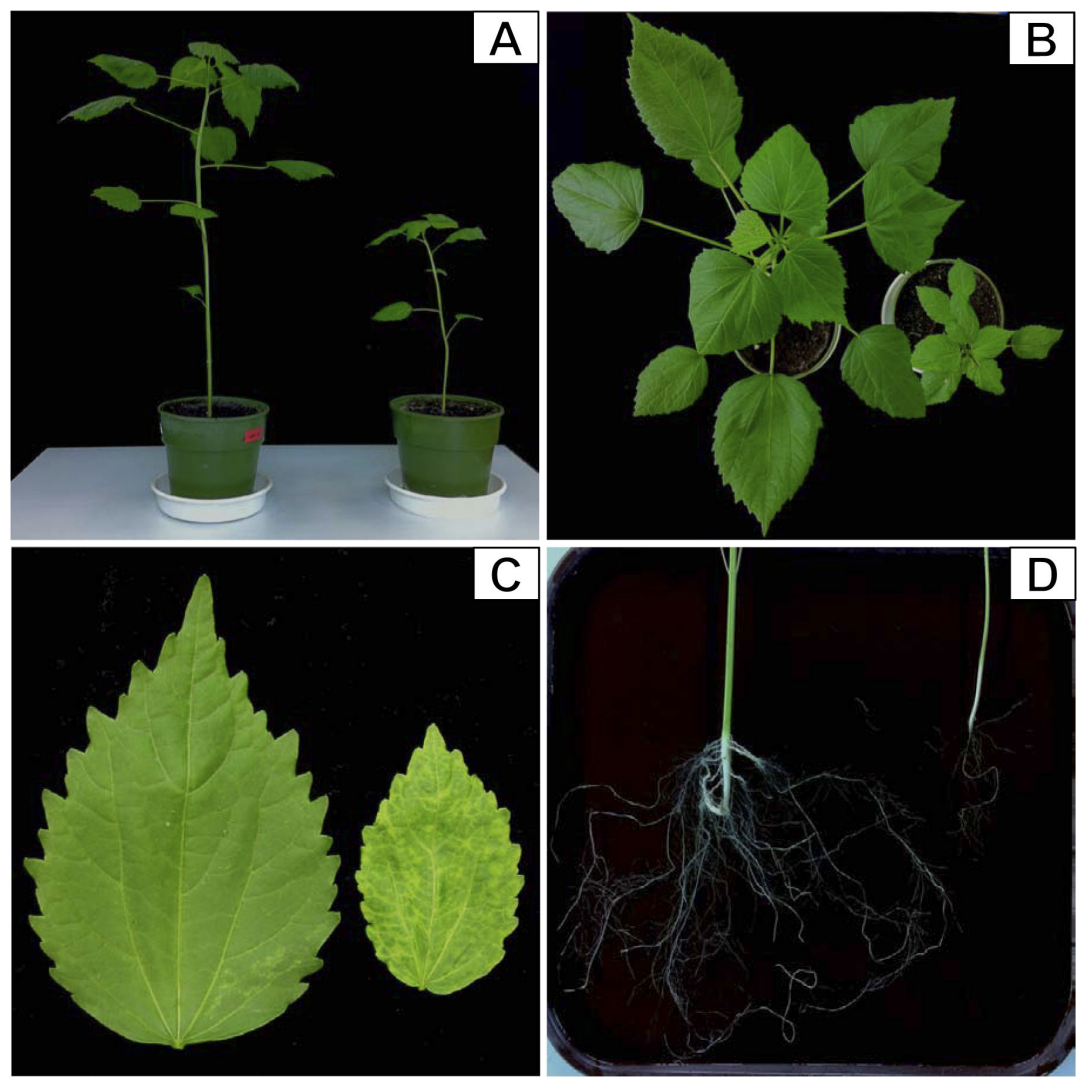
Morphological changes in HCRSV-infected kenaf plants at 15 days post inoculation (dpi). Growth retardation was observed in the HCRSV-infected plants (plant on the right side of each panel; mock plant was displayed on the left). (A) Side view showing plant height. (B) Top view showing plant canopy. (C) Leaf size and (D) Main and adventitious roots. Note the severe reduction in length and branch of HCRSV-infected kenaf roots.

 In this study, the morphological changes of HCRSV-infected kenaf plants were compared with mock plants. Our results showed that the plant height, leaf size and root growth were significantly reduced in HCRSV-infected plants (Figure 1A-D). In particular, development of the main and adventitious roots of kenaf was stunted and less dense, as compared to the mock plant (Figure 1D). The vegetative growth of HCRSV-infected plants was severely retarded (Figure 1).

### 2: Disruption of vascular bundle formation in HCRSV-infected kenaf stem

**Figure 2:**
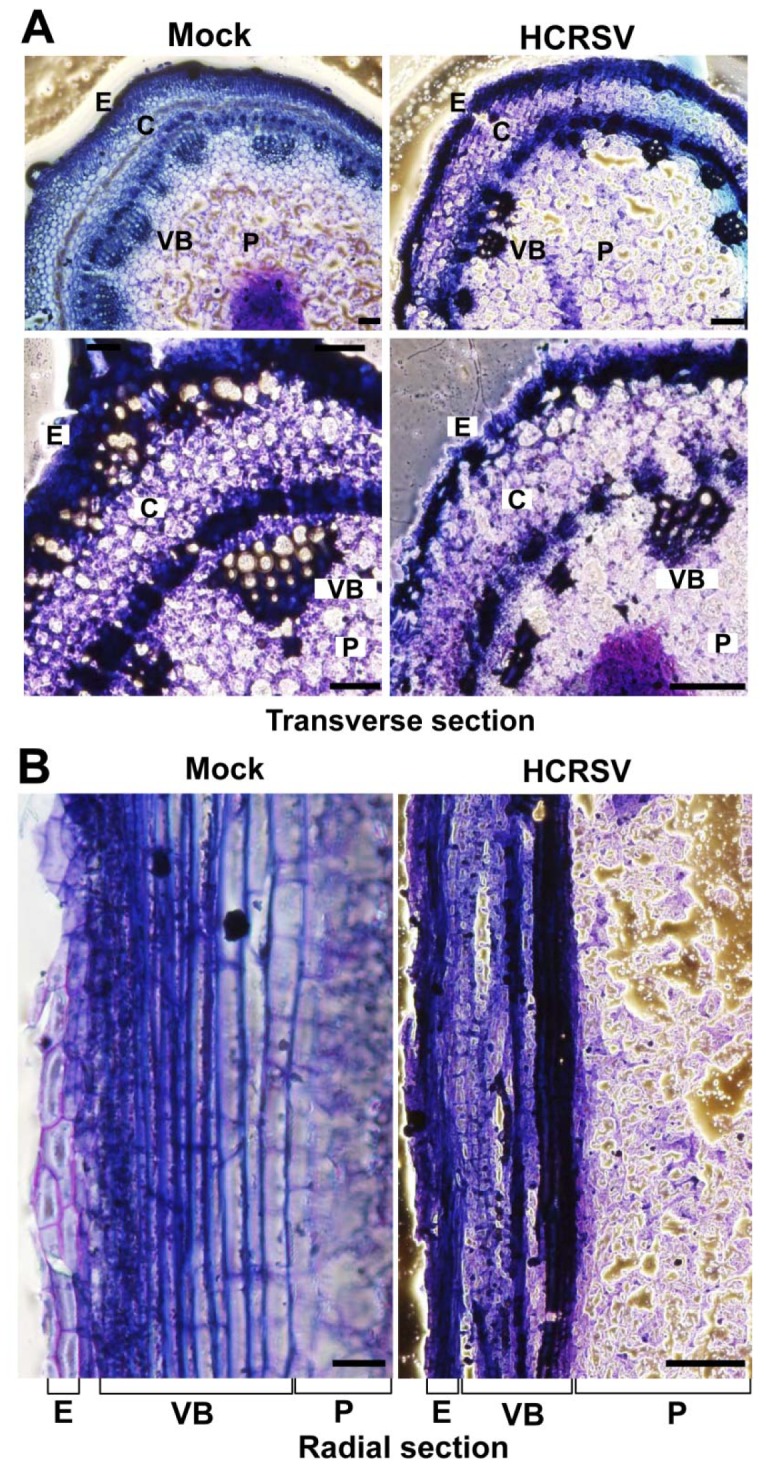
Comparison of transverse and radial sections of mock and HCRSV- infected kenaf stem at 15 dpi. (A) Transverse section of mock and HCRSV-infected kenaf stems. The pith is located in the middle of the transverse section, surrounded by vascular bundles. (B) Partial radial sections of mock and HCRSV-infected kenaf stems. E, epidermis; VB, vascular bundle; C, cortex; P, pith. Bar = 100 µm.

 Transverse and radial sections were analysed using kenaf stems at 15 dpi with HCRSV. Overall the structure of HCRSV-infected stems and the epidermal cells of HCRSV-infected plants were disorganized (Figure 2). The development of vascular bundles including the fiber and the cortex was severely disrupted (Figure 2A) and the vessels appeared to be collapsed (Figure 2B). The brown colored spaces in the pith region were artifacts that resulted from reflection of illumination from the damaged tissues (Figure 2). Detrimental changes in the water- and food-conducting tissue of HCRSV-infected kenaf were most likely related to its reduced growth. As a non-wood fibrous crop, the reduction in growth (fiber yield) of kenaf is of economic importance. 

### 3: Levels of four conserved miRNAs and their target genes fluctuated in HCRSV-infected kenaf

**Figure 3:**
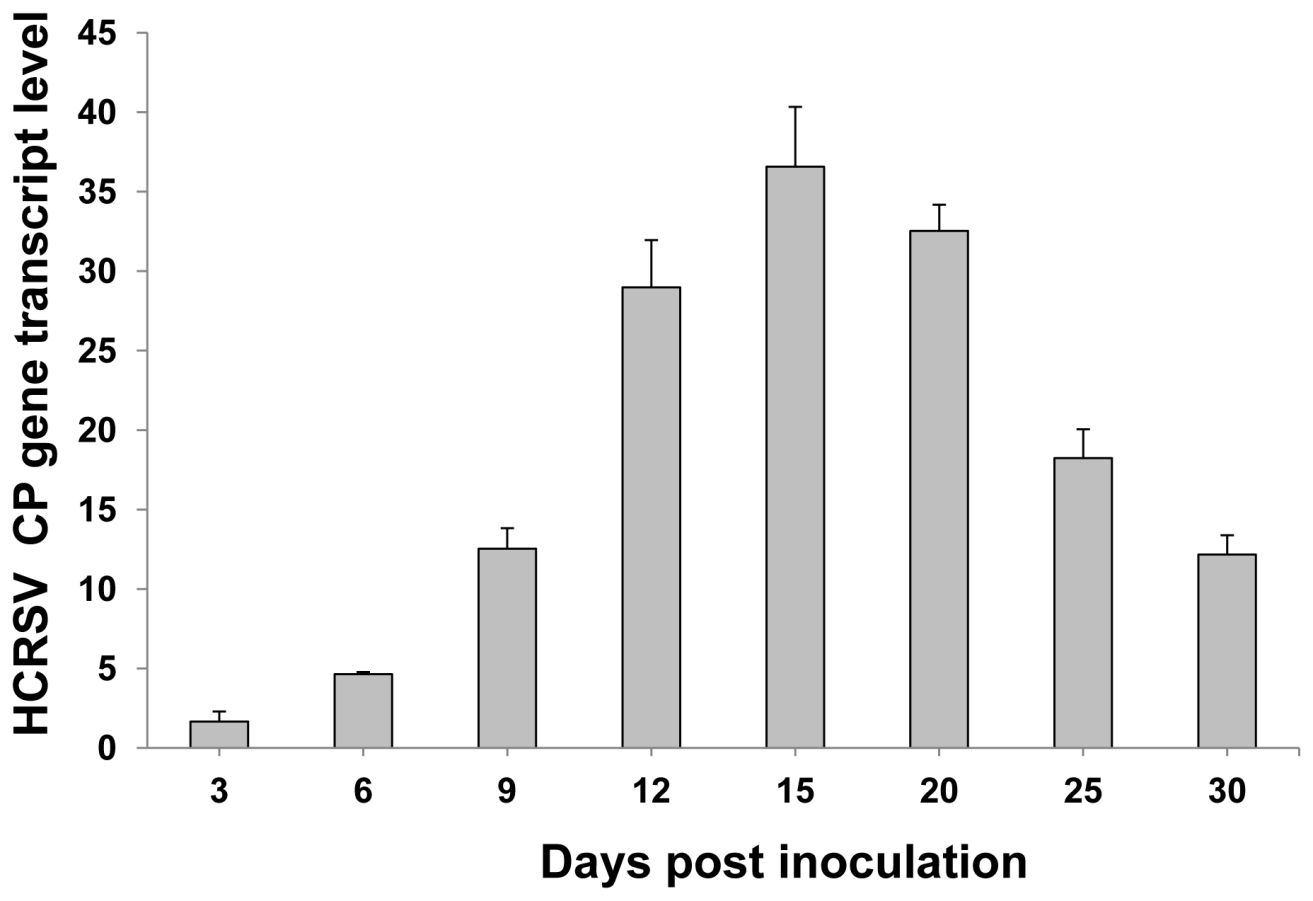
Expression of the HCRSV-CP gene transcript after HCRSV infection as determined by qRT-PCR. The gene transcript level of HCRSV-CP was shown at 3, 6, 9, 12, 15, 20, 25 and 30 days post inoculation (dpi). Relative gene transcript levels (*actin* as internal control) were analyzed using the 2^-∆C^_T_ methods.

**Figure 4:**
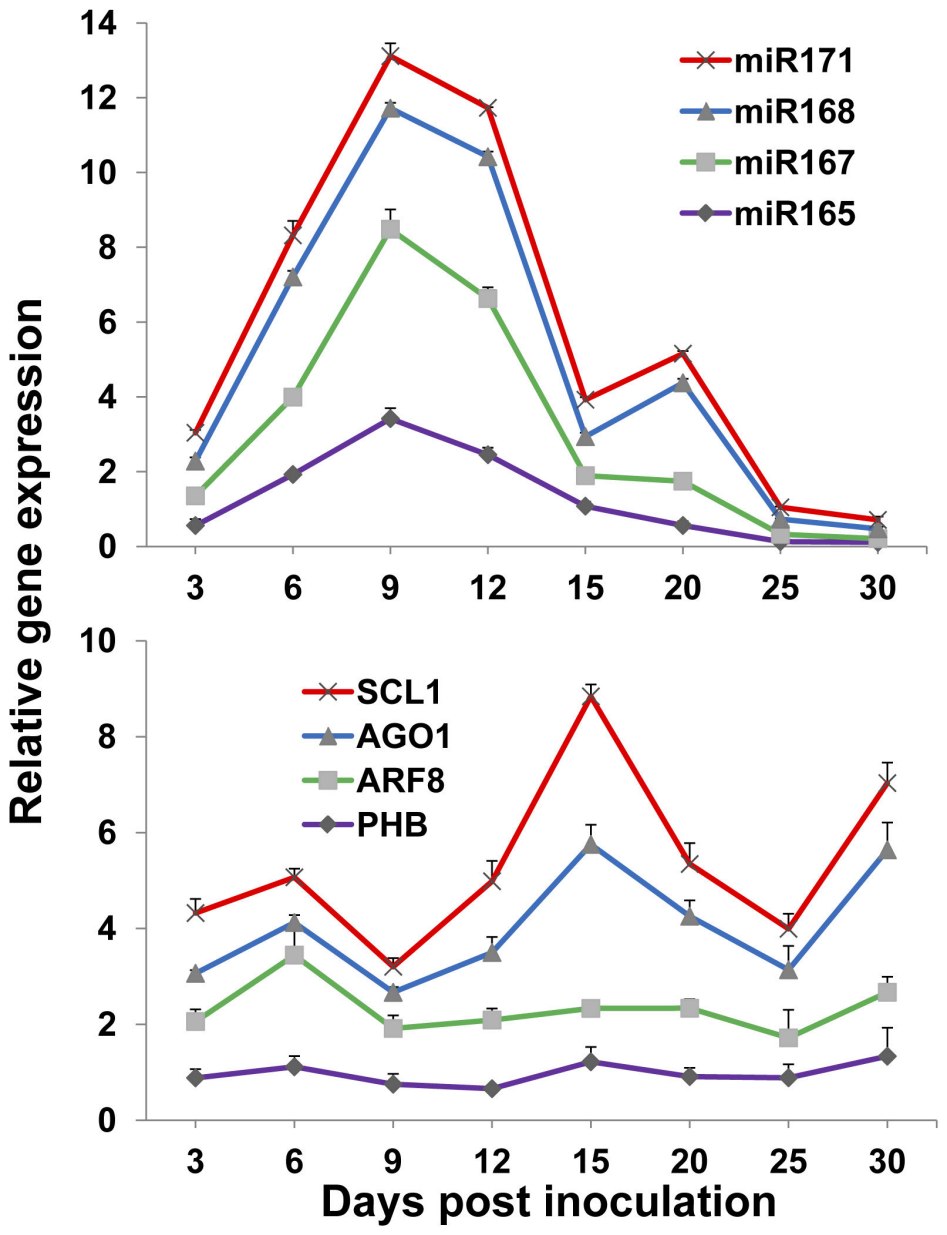
Expression of miR165, miR167, miR168, miR171 and their respective target genes after HCRSV infection as determined by qRT-PCR. The expression levels of miRNAs and their respective target genes were shown at 3, 6, 9, 12, 15, 20, 25 and 30 days post inoculation (dpi). Relative gene transcript levels (*actin* as internal control) were analyzed using the 2^-∆∆C^_T_ methods. The target genes for miR165, miR167, miR168 and miR171 are *phabulosa* (*PHB*), auxin response factor 8 (ARF8), *argonaute 1* (*AGO1*) and *scarecrow-like 1* *protein* (*SCL1*), respectively. The values of miRNAs and their respect target genes in HCRSV-infected plants were calculated by subtracting the values from mock controls which were all set to 1 for standardization. Means of three independent biological repeats were shown with standard deviations.

 The selected plant miRNAs are also conserved in kenaf plants as compared with *Arabidopsis*. The sequences are: miR165: UCGGACCAGGCUUCAUCCCCC; miR167: UGAAGCUGCCAGCAUGAUCUA; miR168: UCGCUUGGUGCAGGUCGGGAA; and miR171: UGAUUGAGCCGCGCCAAUAUC. Plant development was regulated by developmental-related genes during plant growth. Target genes *PHB*, *ARF8* and *SCL1* of miR165, miR167 and miR171 regulate plant leaf, stem and root development. The expression level of these miRNAs and their respective target genes were further investigated in the HCRSV-infected plants within a period of 30 days. Firstly, the gene transcript of HCRSV-CP was investigated to verify the success of HCRSV infection. The results showed that the gene transcript of *HCRSV-CP* was increased after HCRSV infection and reached its highest expression level at 15 dpi and decreased from 20 to 30 dpi (Figure 3). Secondly, different from *HCRSV-CP*, the expression of the four miRNAs and their target genes showed two types of expression. Compared with the mock plants, miR171 and miR168 and their target genes *SCL1* and *AGO1* showed greater fluctuations after HCRSV infection. The transcript levels of these two miRNAs reached the highest peak at 9 dpi, and a second peak at 20 dpi. (Figure 4, upper graph). Their respective target genes *AGO1* and *SCL1* showed opposite expression levels, which reached a peak at 6 dpi and a higher peak at 15 dpi. The expression level of *SCL1* showed the greatest variations, followed by *AGO1* (Figure 4, lower graph). However, even though miR167 and miR165 showed a higher peak at 9 dpi, the expression of their target genes was not significantly affected. These results confirmed that two of the four plant conserved miRNAs targeted the same genes effectively, as reported in other plant species [18-21]. The variations of plant developmental related genes’ expression levels were observed in the HCRSV-infected plants. 

### 4: Upregulation of the transcript level of *AGO1* after *SO* overexpression

 As HCRSV upregulates plant *SO* transcript and increases sulfate levels in kenaf [7] and upregulated *AGO1* in HCRSV-infected plants (Figure 4), the expression level of *AGO1* transcript was further investigated after *SO* overexpression. The efficiency of *SO* overpression and silencing was first verified in the *SO* transient expression leaves (Figure 4A). Interestingly, the transcript level of *AGO1* gene was also upregulated or downregulated (Figure 4B) after *SO* was transiently overexpressed or silenced in kenaf plants. In addition, a full-length *AGO1* gene was cloned from kenaf using the RACE-PCR. The result validated that the full-length of *AGO1* gene from kenaf is 3315 bp and its predicted protein is 121.9 kDa, containing 1104 aa (GenBank accession KF147914). 

### 5: Transcript levels of four selected miRNAs after *SO* overexpression or silencing

 The expression levels of four other selected plant conserved miRNAs (miR171, miR168, miR167 and miR165) were also found to be upregulated or downregulated under *SO* overexpressed or silenced conditions. These results showed that upregulation of *SO* also increased expression levels of several plant conserved miRNAs, demonstrating a correlation between the fluctuations of the four conserved miRNAs and kenaf plants after infection with HCRSV.

## Discussion

 Kenaf plant growth retardation and four selected plant conserved developmental related miRNAs were investigated after HCRSV infection. The four conserved miRNAs (miR165, miR167, miR168 and miR171) and their target genes were previously investigated in CMV- and Tomato aspermy virus (TAV)-infected plants. Although their selected miRNAs (including the four miRNAs used in this study) displayed fluctuations after CMV and TAV infections, the changes of their target mRNAs were comparable, suggesting a similar mechanism in perturbing miRNA pathways [20]. Within 30 days of HCRSV infection, two of the four miRNAs and their corresponding target genes indeed fluctuated notably. Two miRNAs (miR171 and miR168) reached the highest expression levels around 10 dpi and dropped until 30 dpi. Their target genes displayed opposite expression patterns corresponding to their regulating miRNAs. Similar expression profiles were also observed in CMV- and TAV-infected plants [20]. On the other hand, the other two miRNAs miR165 and miR167 and their respective target genes displayed less significant changes. Since different miRNAs play different regulation roles during plant development, we did not expect the similar expression profiles for each miRNA. In addition, the regulation between miRNAs and their target genes are a multi-regulation process, meaning one miRNA can target many genes and one gene can be targeted by many miRNAs. It is reasonable that some miRNAs will display different expression profiles after HCRSV infection. 

 AGO1 is involved in the miRNA pathway which plays essential roles in regulating plant development. The gene transcript of *AGO1* was upregulated after *SO* was transiently overexpressed or in HCRSV-infected kenaf. In this study, for the first time, we have obtained the full-length sequence of *AGO1* from kenaf. The fluctuations of the two selected miRNAs miR168 and miR171, together with elevated level of *AGO1*, regulate the respective target genes. Co-regulation of miR168 and *AGO1* gene and stabilization of miR168 by *AGO1* are two regulatory processes required to maintain AGO1 at a certain level, and disrupting any of these regulatory processes disturbs a proper functioning of the miRNA pathway [25]. Among the four conserved plant development-related miRNAs, miR168 displayed more significant fluctuation. AGO1, the target gene of miR168, is the main component in the RISC and displayed corresponding fluctuations. The feedback regulation between miR168 and AGO1 causes other related miRNAs and their target genes fluctuations. Because the development-related genes were affected, the plant growth was retarded. 

**Figure 5:**
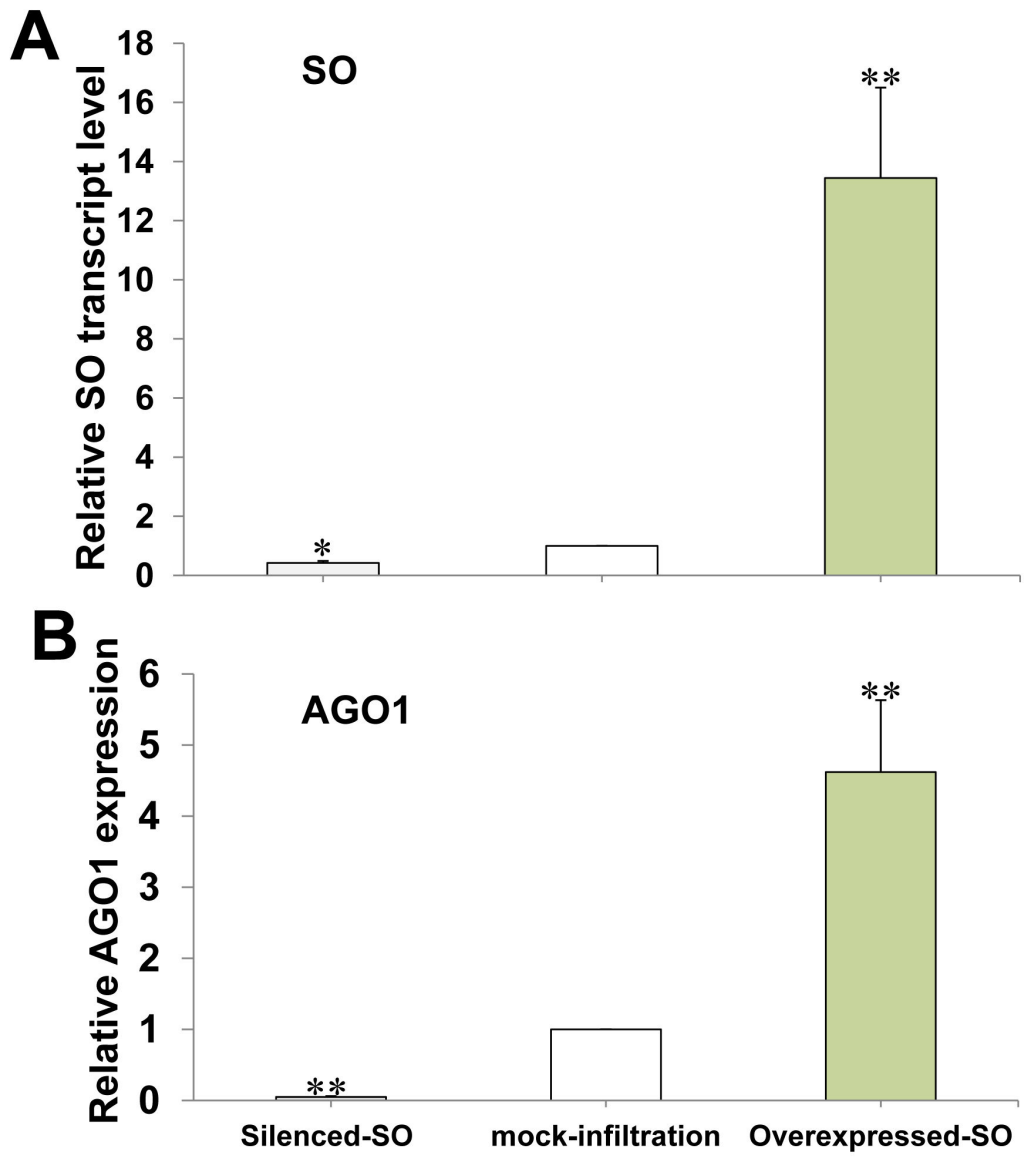
Upregulation of *AGO1* after SO overexpression. (A) Relative SO gene transcript level was used to test the SO gene overexpression and silencing efficiency. (B) Upregulation or downregulation of *AGO1* gene transcript after SO overexpression or silencing. Actin gene was used as internal control. The relative gene expression amount was analyzed using the 2^-∆∆C^_T_ method. Significant differences among silenced-SO, mock-infiltration and overexpressed-SO were calculated using the one sample Student’s *t*-test analysis. Asterisks (* and **) indicate significance at 0.05 and 0.01 levels of confidence, respectively.

**Figure 6:**
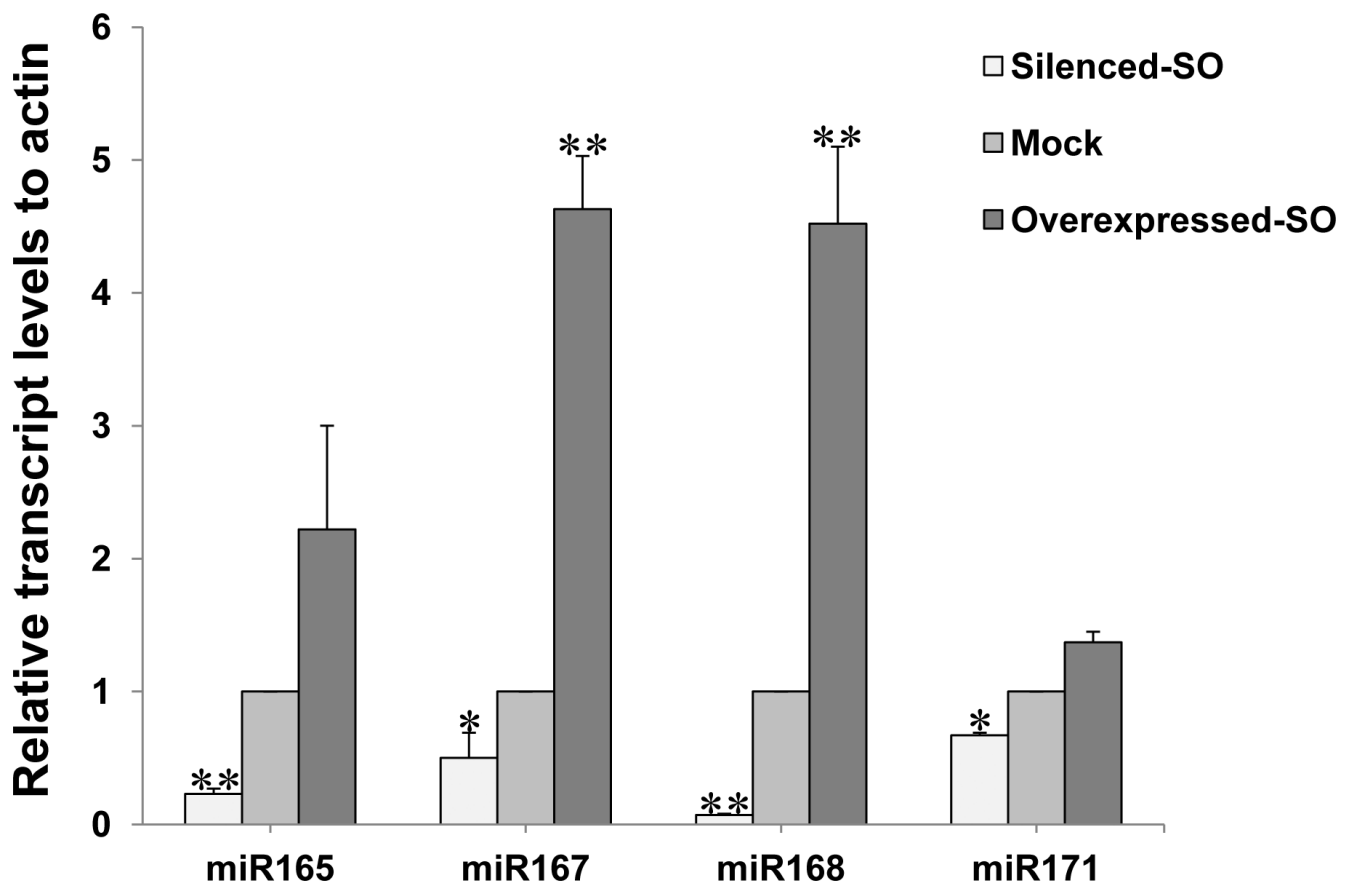
Upregulation or downregulation of plant conserved miRNAs (miR165, miR167, miR168 and miR171) expression levels at 3 dpi of SO overexpressed or silenced leaves as determined by qRT-PCR. Relative miRNAs gene transcription levels (actin as internal control) were analyzed using 2^-∆∆C^_T_ method. The value of mock control was subtracted from the overexpressed or silenced HcSO samples. Means of three independent biological repeats were shown with standard deviations. Significant differences between mock and Agroinfiltrated leaves were calculated using the one sample Student’s *t*-test. Asterisks (* and **) indicate significance at 0.05 and 0.01 levels of confidence, respectively.

 The differences of the miRNA fluctuation in HCRSV-infected kenaf leaves and Agro-infiltrated kenaf leaves may be due to the differences of *in vitro* and *in vivo* systems. In both *SO* overexpressed/silenced kenaf leaves and HCRSV-infected kenaf, miR168 and its target gene *AGO1* showed significant change profiles (Figures 4 to 6). This suggests that miR168 plays a crucial role during HCRSV infection. After HCRSV infection, miR171 and its target gene displayed the greatest changes compared with the mock plants. The variations between the mock and HCRSV-infected plants indicate the severe growth retardation of the HCRSV-infected plants (Figure 1D). The root is the crucial organ for plants to absorb and transport water and nutrients which contribute to the growth and development of the upper portion of the plants. Disrupting the normal root development of HCRSV-infected plants, the development of the whole kenaf including stem and leaf was severely inhibited. 

 This study showed that the plant development-related miRNAs fluctuated after HCRSV infection, which displayed plant growth retardation. This analysis of plant development-related miRNAs fluctuations and plant growth retardation may help to improve the understanding of plant viral infection and present strategies to prevent infection in future. For example, the fluctuation of miR168 can influence the fluctuation of AGO1 which is the main component in RNA-induced silencing complex that functions to prevent infection. However, we have not yet established the strong link to correlate miRNAs fluctuations and plant growth retardation after virus infection. A further study needs to be performed in subsequent research. 

 In conclusion, kenaf plant growth was severely retarded and four selected plant conserved miRNAs were found to fluctuate after HCRSV infection over time. The fluctuations of certain miRNAs, which target conserved plant development related genes, imply their indirect linkages to the plant growth retardation observed in HCRSV-infected kenaf. Since kenaf is a non-wood fiber-producing crop, the reduction in fiber yield resulting from infection by HCRSV is detrimental to its production and reduces its economic values. 
